# Supplementary material for: Phloretin Transfersomes for Transdermal Delivery: Design, Optimization, and In Vivo Evaluation
Source: Molecules. 2023 Sep 24;28(19):6790. doi: 10.3390/molecules28196790 (PMC10574780; doi:10.3390/molecules28196790)
Supplement: Supplementary file 1 [file molecules-28-06790-s001.zip › molecules-2584834-supplementary.pdf]

Phloretin Transfersomes for Transdermal Delivery: Design, Optimization, and In Vivo Evaluation

Jiawen Wang [1], Yuanyuan Zhao [2], Bingtao Zhai [1], Jiangxue Cheng [1], Jing Sun [1], Xiaofei Zhang [1], Dongyan Guo [1]*

1State Key Laboratory of Research & Development of Characteristic Qin Medicine Resources (Cultivation), and Shaanxi Key Laboratory of Chinese Medicine Fundamentals and New Drugs Research, and Shaanxi Collaborative Innovation Center of Chinese Medicinal Resources Industrialization, Shaanxi University of Chinese Medicine, Xi'an 712046, China

2 Yulin Hospital of Traditional Chinese Medicine, Yu lin 719000, China

*Correspondence: Dongyan Guo

E-mail: xmc2051080@163.com Tel: 0086-029-38185180

Methodological Investigations of HPLC

Preparation of the control solution

Precisely weigh 10 mg of the phloretin control product in a 10 mL volumetric flask and dilute with methanol to the scale. Abbreviations will be explained when first used. Next, transfer 2 mL of the solution into a 10 mL volumetric flask to prepare a 200 $\mu g \cdot mL^{-1}$ solution of phloretin control.

Preparation of Phl-TFs test solution

Precisely measure 0.5 mL of Phl-TFs suspension and place it in a 10 mL volumetric flask. Dilute the mixture with chromatography methanol to the appropriate scale. Emulsify the mixture using ultrasonic technology for 10 min. Filter the mixture through an organic filter with 0.22 μm openings. Store the mixture in the refrigerator at 4°C for later use.

Specificity

To investigate the specificity of the method, 10 μL of TFs blank solution, phloretin control solution, and Phl-TFs solution were taken and detected under HPLC conditions. The results are shown in Figure S1, which indicates that under the chromatographic conditions, the peak shape of phloretin is good and there is no impurity peak, the chromatographic conditions have good specificity and high sensitivity, and the retention time of the peak of phloretin is about 6.4 min.

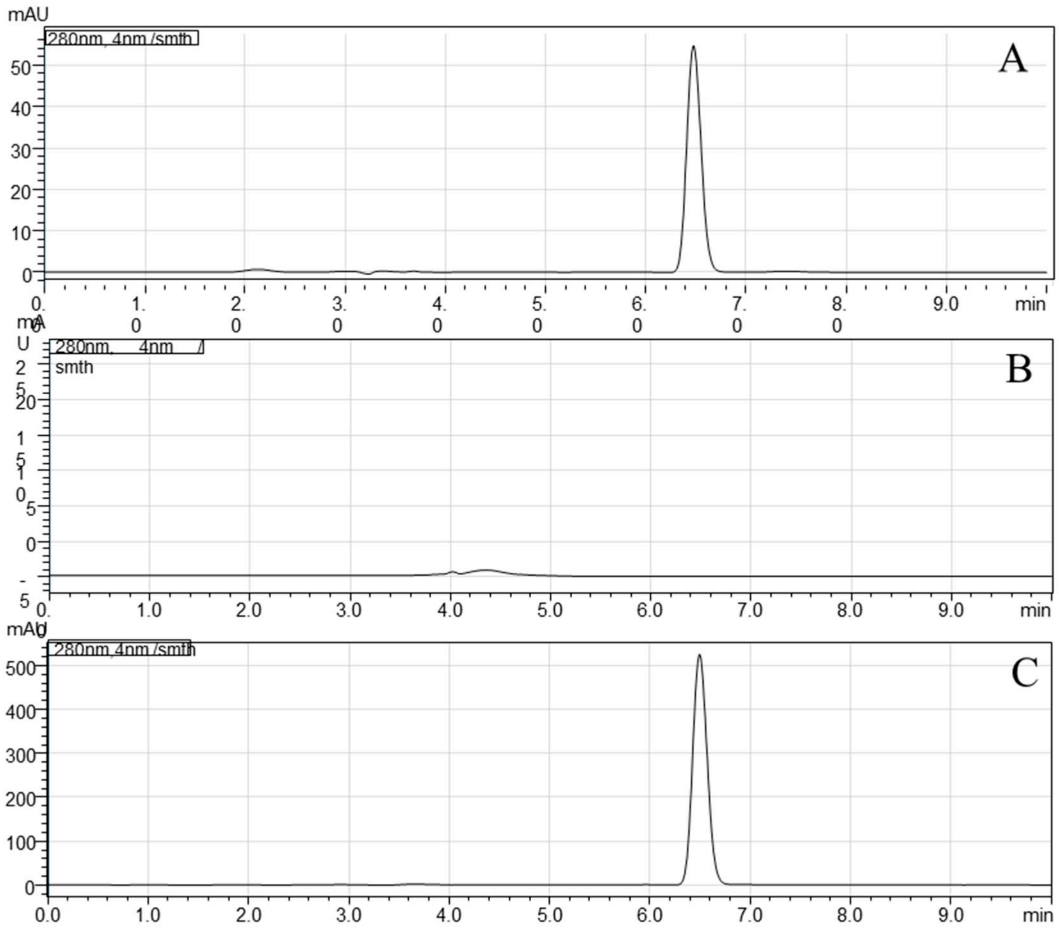

Figure S1 HPLC of phloretin

(A) Phl-TFs solution. (B) TFs blank solution. (C) phloretin control solution.

Standard curve

Precisely take up phloretin control solution, add methanol to dilute into a series of phloretin mass concentration of 10, 20, 40, 80, 120, 160, 200 μg·mL^{-1}, according to the chromatographic conditions in the detection, the phloretin peak area (Y) as the vertical coordinate, the mass concentration (X) for the horizontal coordinate of the standard curve, Y = 55469 X - 21878 (r = 0.9997). The standard curve was plotted with the peak area (Y) as the vertical coordinate and the mass concentration (X) as the horizontal coordinate, and the result was Y = 55469 X - 21878 (r = 0.9997), which is shown in Figure S3, the results showed that the linear relationship between peak area and mass

concentration of phloretin was good in the range of 10.00-200.00 μg·mL^{-1}.

Precision

The phloretin control solution was taken up accurately following the chromatographic conditions. The injection was repeated 6 times and the peak area determination was recorded to calculate its content. The RSD was calculated to check the method's accuracy. The results are presented in Table S1

Stability

Exactly 1 mL of the test solution was aspirated and the phloretin concentration was measured at 0, 2, 4, 6, 8, 10 and 12 h. The stability of the samples was examined over a period of 12 h. The results are presented in Table S1.

Repeatability

6 samples from the same batch were taken to prepare phloretin samples and the concentration of phloretin was determined according to the chromatographic conditions. The results are presented in Table S1.

Table S1 Methodological Investigations table（n=6）

Methodological Investigations	average value（μg·mL^{-1}）	RSD（%）
precision	103.74	0.51
stability	49.65	0.19
repeatability	49.75	0.18

Sample recovery rate

Precisely measure 1.00 mL of the test solution and 0.50 mL of the control solution, and prepare 3 parallel copies. Determine the content of phloretin and calculate the spiked recoveries. These findings demonstrate the accuracy and reliability of the methodology. The results are presented in Table S2 revealing an average recovery of

96.28% with an RSD of 0.95%.

Table S2 Sample Recovery Table（n=6）

Sample content (μg)	Amount of control added (μg)	Measured quantity (μg)	Recovery rate (%)	Average recovery (%)	RSD (%)
49.75	50.00	95.30	95.54	96.28	0.95
49.75	50.00	97.38	97.62		
49.75	50.00	95.35	95.59		
49.75	50.00	96.18	96.42		
49.75	50.00	95.20	95.44		
49.75	50.00	96.80	97.04		

Table S3 Box-Behnken Design and Results

Run	A	B	C	EE（%）
1	2.5	1.25	45.00	90.46
2	1	1.25	15.00	91.34
3	4	2.00	45.00	89.29
4	2.5	0.50	15.00	81.88

Table S4 Analysis of variance

Source	Sum of Squares	df	Mean Square	*F* Value	*p*-value Prob > F	
Model	344.05	9	38.23	5.68	0.0160	significant
A- SPC / CHOL (w/v)	0.38	1	0.38	0.056	0.8190	
B- Phloretin（mg/mL）	18.09	1	18.09	2.69	0.1451	
C- Hydration time (min)	0.71	1	0.71	0.11	0.7542	
AB	3.16	1	3.16	0.47	0.5148	
AC	3.01	1	3.01	0.45	0.5250	
BC	3.10	1	3.10	0.46	0.5188	
A^2	11.53	1	11.53	1.71	0.2318	
B^2	248.91	1	248.91	37.00	0.0005	
C^2	34.78	1	34.78	5.17	0.0571	
Residual	47.09	7	6.73			
Lack of Fit	23.44	3	7.81	1.32	0.3843	not significant
Pure Error	23.65	4	5.91			

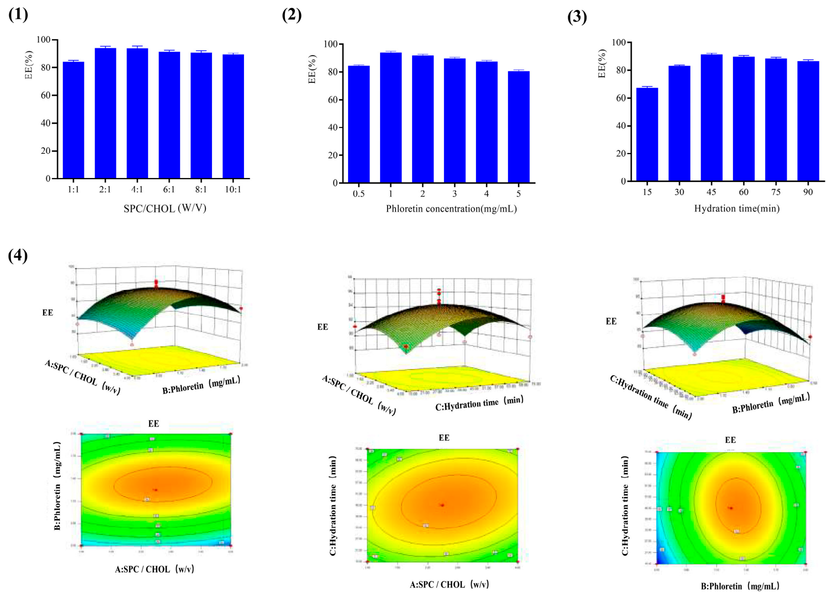

Figure S2 Single-factor and BBD-RSM examination Phl-TFs.

(1) Ratio of SPC to CHOL. (2) Phloretin concentration. (3) Hydration time. (4) Three-dimensional effect surface plots and two-dimensional plots of EE.

Table S5 Analytical design factor level table

level	ultrasound time (min)	ultrasound power (W)
-1	4	135
0	5	180
1	6	225

Table S6 Experimental design and results

Experimental combinations	EE (%)	VS (nm)	ZP (-mV)	PDI	OD
A1B1	85.53	121.15	20.12	0.308	0.00
A1B2	88.42	148.20	19.23	0.282	0.00
A1B3	87.26	110.83	20.85	0.311	0.35
A2B1	87.61	106.25	20.18	0.308	0.29
A2B2	88.15	91.37	19.82	0.257	0.51
A2B3	86.39	112.45	19.50	0.322	0.02
A3B1	86.85	134.08	20.35	0.372	0.01
A3B2	89.04	145.75	20.17	0.377	0.00
A3B3	86.32	153.78	20.40	0.364	0.00

Table S7 **Analysis of variance (ANOVA) table**

Source of variance	Sum of squares	Degree of freedom	Mean Square	F	*p*
ultrasound time	0.110	2	0.055	3.180	< 0.05
ultrasound power	0.008	2	0.004	1.134	< 0.05
Interaction	0.195	4	0.049	0.078	< 0.05
Error	0.305	1			
Total	0.618	9		-	-
